# Comparative High-Density Linkage Mapping Reveals Conserved Genome Structure but Variation in Levels of Heterochiasmy and Location of Recombination Cold Spots in the Common Frog

**DOI:** 10.1534/g3.116.036459

**Published:** 2016-12-28

**Authors:** Gemma Palomar, Freed Ahmad, Anti Vasemägi, Chikako Matsuba, Alfredo G. Nicieza, José Manuel Cano

**Affiliations:** * Research Unit of Biodiversity (University of Oviedo-Consejo Superior de Investigaciones Científicas-Principado de Asturias), University of Oviedo, 33600 Mieres, Asturias, Spain; † Department of Biology of Organisms and Systems, University of Oviedo, 33006, Asturias, Spain; ‡ Department of Biology, University of Turku, 20014, Finland; § Department of Aquaculture, Institute of Veterinary Medicine and Animal Science, Estonian University of Life Sciences, 51006 Tartu, Estonia

**Keywords:** linkage map, *Rana temporaria*, Spanish lineages, recombination cold spots, heterochiasmy

## Abstract

By combining 7077 SNPs and 61 microsatellites, we present the first linkage map for some of the early diverged lineages of the common frog, *Rana temporaria*, and the densest linkage map to date for this species. We found high homology with the published linkage maps of the Eastern and Western lineages but with differences in the order of some markers. Homology was also strong with the genome of the Tibetan frog *Nanorana parkeri* and we found high synteny with the clawed frog *Xenopus tropicalis*. We confirmed marked heterochiasmy between sexes and detected nonrecombining regions in several groups of the male linkage map. Contrary to the expectations set by the male heterogamety of the common frog, we did not find male heterozygosity excess in the chromosome previously shown to be linked to sex determination. Finally, we found blocks of loci showing strong transmission ratio distortion. These distorted genomic regions might be related to genetic incompatibilities between the parental populations, and are promising candidates for further investigation into the genetic basis of speciation and adaptation in the common frog.

Intra- and interspecific comparative genomic studies shed light on basic evolutionary processes such as sexual differentiation, adaptation, and speciation (Koonin *et al.* 2000; Kocher 2004; Nadeau and Jiggins 2010; Bachtrog 2013). While comparative genomic studies have mostly focused on model organisms, such as humans and mice (*e.g.*, Bernstein *et al.* 2005), the advances in next-generation sequencing technology have facilitated genomic analysis in species lacking a reference genome (Kocher 2004; Miller *et al.* 2007; Gagnaire *et al.* 2013; Brelsford *et al.* 2016a). Amphibians are good models for comparative genomics due to their extraordinary biodiversity, worldwide distribution, and wide range of variation in genome size and karyotype (Duellman and Trueb 1986; Green and Sessions 1991). In particular, the common frog, *Rana temporaria*, has a wide distribution range across contrasting environmental conditions and very well reported cases of local adaptation (Miaud *et al.* 1999; Laurila *et al.* 2002; Laugen *et al.* 2003; Cano *et al.* 2004; Phillimore *et al.* 2010).

Phylogenetic analyses of the common frog have revealed multiple, deeply diverged evolutionary lineages or clades across Europe (Palo *et al.* 2004; Vences *et al.* 2013). There are two widely distributed Western and Eastern clades, which separated ∼0.7 MYA (million years ago) (Palo *et al.* 2004), and whose contact zone is in Central Europe (Schmeller *et al.* 2008; Teacher *et al.* 2009; Vences *et al.* 2013). In northwest Spain, another divergent evolutionary lineage, frequently referred to as subspecies *R. t. parvipalmata* (Seoane 1885), has been identified. This lineage is basal (*i.e.*, divergence 1.12 MYA) to the Western and Eastern clades (Veith *et al.* 2002, 2003; Palo *et al.* 2004). In addition, there is evidence for at least one other lineage, a sister group of *R. t. parvipalmata*, also located in northern Spain (Vences *et al.* 2013).

Earlier linkage maps of *R. temporaria* have been constructed using individuals from the Western and Eastern clades (Cano *et al.* 2011; Rodrigues *et al.* 2013, 2016). The Western and Eastern lineage maps showed different locus order that might indicate potential genomic rearrangements (*e.g.*, in linkage groups Xt3, Xt5, Xt6, and Xt7B), which might be linked to adaptive processes (Lee 2002). However, we still lack a comprehensive understanding of the genomic differences among lineages of the common frog, and especially about the genome structure of the early diverged lineages in the north of the Iberian Peninsula.

The aim of the present study is to compare recombination patterns, synteny, and putative sex-linked chromosomes of the Iberian lineages with more recently diverged lineages of the common frog. To accomplish these objectives, we used Restriction site-Associated DNA sequencing (RAD-seq) and microsatellite markers to construct a high-density consensus linkage map of *R. temporaria*, based on a cross between parents from the southwestern end of the species range in the north of the Iberian Peninsula.

## Materials and Methods

### Mapping family and DNA extraction

This study is based on a large full-sib family from a single *R. temporaria* cross. Adults were sampled in two localities from the North of Spain (Bárcena Mayor, Cantabria, N 43.13103 W 4.17879; and Vega de Candioches, León, N 42.99910 W 5.92124). We crossed a male from Candioches [high altitude: 1687 m.a.s.l. (metres above sea level)] and a female from Bárcena (low altitude: 551 m.a.s.l.) belonging to two different mtDNA clades (Vences *et al.* 2013; A. G. Nicieza, unpublished data). Frogs from these localities are exposed to contrasting environmental conditions (*i.e.*, highland with short growing season and cold winters *vs.* lowland with long growing season and mild winters). The controlled cross was carried out in the laboratory of the Amphibian Facilities at the University of Oviedo. Female and male abdomens were pressed gently by hand to obtain eggs and sperm without harming the animals. Tadpoles were individualized at Gosner stage 25 and were kept at 12 hr light:12 hr dark photoperiod and 14° until they reached Gosner stage 42. Then, they were killed with an overdose of benzocaine and frozen. DNA from the brains of parents and 184 F1 offspring was extracted with the DNeasy Blood and Tissue Kit (QIAGEN). DNA quality was assessed via 0.7% agarose gel electrophoresis and DNA concentration was determined with a Qubit fluorometer. DNA extractions were diluted to 100 ng/μl for subsequent library preparation.

### RAD-seq

Two libraries were prepared according to the slightly modified protocol of Elshire *et al.* (2011) (Supplemental Material, File S1). Briefly, DNA was digested with restriction enzymes *Pst*I, and *Bam*HI and the fragments of each individual were ligated to one of the 94 modified Illumina adapters, which contain barcode sequences, with T4 DNA ligase. Adapter-ligated DNA was combined to two library pools consisting of 92 offspring and the two parents in each pool. After purification, DNA fragments of a certain size (300–600 bp range) were selected in each library using an E-Gel iBase Power System as in Pukk *et al.* (2015). Size-selected fragments were amplified by a PCR using 18 cycles and purified with a QIAquick PCR purification kit (QIAGEN). An Agilent 2100 Bioanalyzer system was used to check the quality and quantity of size-selected and amplified libraries. At the end, fragments of average 400 bp length (80% of fragments with size range 300–550 bp) from the two libraries were sequenced on two paired-end lanes (2 × 100) with the Illumina HiSequation 2000 in the Illumina Genome Analyzer platform at the Center for Genomic Regulation in Barcelona, Spain.

### Genotyping

Raw Illumina reads with low quality were discarded as well as reads with ambiguous barcode sequences. As the forward and reverse reads did not overlap, forward reads were used for the first part of the analysis. We demultiplexed the sequences and barcodes were trimmed as a result of this process. Low quality ends were discarded resulting in 91 bp reads. Because of the lack of a reference genome for *R. temporaria*, the reads from the presumably heterogametic parent (*i.e.*, sire) were used to generate the reference sequence. To that end, identical reads were first collapsed with FASTQ/A Collapser of the FASTX-toolkit (Gordon and Hannon 2010). Second, the remaining sire’s sequences were clustered with CD-HIT EST (Li 2015) at 90% of similarity. An assembly *de novo* was performed with the resulting contigs in the MIRA assembler (Chevreux 2005) and a sire-based reference sequence was obtained for each contig. Third, the reads of the dam and sire were aligned to this reference sequence using the software Bowtie2 (Langmead and Salzberg 2012). The dataset of forward read contigs was obtained after discarding contigs mapped with < 10 reads or > 1000 reads for each parent, as well as those with > 10% of mismatch. We used the information from the forward read to select the corresponding reverse read and create a dataset for both forward and reverse read contigs. Fourthly, to select informative loci, we conducted parental SNP calling with the software Samtools (Li *et al.* 2009). A heterozygotic genotype was called when the minor allele frequency (MAF) among reads was > 0.1. Uninformative SNPs (*i.e.*, both parents with alternative homozygous genotypes) were discarded. Finally, the selected SNPs were used to map the progeny reads. Following DaCosta and Sorenson (2014) and minimizing the mismatching, contigs mapped with < 200 or > 20,000 total reads were rejected.

SNP-calling was performed again with a quality threshold of 100 and a maximum of four SNPs allowed per contig. Parents and progeny were deemed heterozygotes if the MAF was > 0.1 and SNPs were excluded if > 25% of progeny genotypes were missing and > 6% of progeny showed noncompatible genotypes. SNPs showing segregation distortion were not included in the initial linkage maps (*X^2^* test, *p* < 0.05). However, to later determine potentially incompatible regions between the two lineages, we retained loci showing relatively mild segregation distortion (*X^2^* test, *p*-value 0.05–0.005) for construction of new linkage maps and permutation analysis (see *Transmission ratio distortion*). Genotyping error was calculated for both sire and dam by comparing replicated genotype calls obtained from two separate sequencing lanes.

### Microsatellites

In addition to the SNPs, microsatellite markers were also included to enable direct comparison with the previous maps (Cano *et al.* 2011; Rodrigues *et al.* 2013). From 116 tested markers (File S3), 113 microsatellites were successfully amplified within 19 multiplex reactions following the protocol of the QIAGEN Multiplex PCR Master Mix (2 ×) at half volume (25 μl). Amplification reactions started with an initial polymerase activation step at 95° for 15 min, followed by 40 cycles of: 94° for 30 sec, 55° (for the markers: Rib01, Rib 06, Rib 08, and Rib15) or 60° (for the rest) for 90 sec, and 72° for 60 sec; and finally, an extension stage of 60° for 30 min. Microsatellite analysis was performed using an ABI 3100 automatic DNA Sequencer.

Genotypes were determined using the software GENEMARKER v 2.4 (Soft Genetics, State College, PA). Uninformative microsatellites, as well as those with null alleles, were excluded from subsequent analysis (File S3).

As for the SNPs from the RAD-seq, microsatellites showing Mendelian segregation violations (*X^2^* test, *p* < 0.05) were removed before the linkage analysis.

### Map construction

Markers, both SNPs and microsatellites, were assigned according to their segregation pattern to five categories: *nn*x*np*, *lm*x*ll*, *ef*x*eg*, *abxcd*, and *hk*x*hk*. Sex-specific maps were constructed from informative markers of each sex (*nn*x*np* and *lm*x*ll*) using the cross type “doubled haploid” (DH) and Maximum Likelihood algorithm in MSTmap (Wu *et al.* 2008). This algorithm has been shown to be more successful at ordering the loci compared to other methods, such as weighted least squares and minimum sum of adjacent recombination fractions (Hackett and Broadfoot 2003). The Kosambi mapping function was used to calculate the genetic distance between markers and a *p*-value of 1 × 10^−8^ was used as the threshold for clustering the markers into the linkage groups. Due to the lack of linkage phase information, the dataset was duplicated, changing the phase of each marker (Gadau *et al.* 2001; Brelsford *et al.* 2016a). Duplicated linkage groups were eliminated manually in the output. As a conservative approach, we used the option “detect bad data” in the software. In addition, possible double crossovers were transformed to unknown (*U*) iteratively followed by an additional round of linkage map reconstruction, until no possible double crossover was found.

The average linkage map was created using the option appropriate for an outbred full-sib family (cross pollinator, CP) in Joinmap 4.1 (Van Ooijen 2011). Identical loci were excluded to decrease computational time and added again after map construction. Linkage groups were identified with a LOD threshold of eight. Small linkage groups (*i.e.*, < 4 markers) were excluded from further analysis. The order of the markers within each linkage group was determined with the Maximum Likelihood mapping algorithm, which assumes no crossover interference. Therefore, the distance between markers was calculated using the Haldane mapping function. Spatial sampling was used with five thresholds: 0.1, 0.05, 0.03, 0.02, and 0.01. Three map optimization rounds were run in each spatial sample, using the following parameters: chain length was 10,000, cooling control parameter was 0.0001, and the rounds were stopped after 100,000 chains without improvement. Finally, for the multipoint estimation of recombination frequencies, we used a burn-in of 100,000, five cycles of Monte Carlo Expectation Maximization (chain length per cycle: 100,000), and six sampling periods for recombination frequency. Stabilization of the recombination frequencies was monitored with the sum of recombination frequencies of adjacent fragments and the mean number of recombination.

The name of the linkage groups follows the *Xenopus tropicalis* homology. The expected genome length and observed genome coverage were calculated according to Chakravarti *et al.* (1991) and Cervera *et al.* (2001), respectively. The observed length (*G*_O_) was the sum of the observed length of the linkage groups. The expected length (*G*_E_) was the sum of the expected length of the linkage groups, which was calculated multiplying the observed length of each linkage group by the factor (*m* + 1) / (*m* − 1), *m* being the number of markers of the linkage group. The observed genome coverage, understood as the proportion of the genome comprised in our recombination map, was the ratio *G*_O_/*G*_E_.

### Transmission ratio distortion markers

Earlier studies have demonstrated that excluding markers that show segregation distortion from linkage mapping may result in the exclusion of certain chromosome regions from the map (Cervera *et al.* 2001; Doucleff *et al.* 2004). However, a large number of distorted markers may also increase the chance of type I errors and may result in inaccurate estimation of genetic distances (Cervera *et al.* 2001). Therefore, we included only markers with moderate levels of segregation distortion (*X^2^*, *p*-values from 0.05 to 0.005) in the linkage analysis. None of the markers caused big gaps (*i.e.*, >50 cM) of recombination distance. To identify distorted regions, instead of only distorted markers, we used a kernel smoothing and permutation test (for details see Bruneaux *et al.* (2013) and Ozerov *et al.* (2016)). By taking into account differences in marker density, this strategy increases the statistical power for detecting regions where several adjacent markers show high distortion. For this analysis, we ran one million permutations in the R software v. 3.0.2 (R Core Team 2013).

### Analysis of the homology

The genomes from *X. tropicalis* (version 9.0, xenbase.org) and *Nanorana parkeri* (version 2, gigadb.org) were used to evaluate the homology and the synteny among amphibian genomes. References from both forward and reverse sire contigs containing SNPs of the linkage map were aligned to a draft genome of *R. temporaria* (File S2) using Bowtie2 (Langmead and Salzberg 2012). The draft scaffolds were selected when forward and reverse references aligned within 600 bp from each other because, during library construction, the size of the fragments was <550 bp. This allowed the use of longer sequences for interspecific comparisons, increasing the power of the homology searches. We searched for homology in NCBI Nucleotide (http://ncbi.nlm.nih.gov) and Swissprot (http://uniprot.org) databases as well as in *X. tropicalis* and *N. parkeri* genomes using blast. Homologous sequences of other species were retained if the best hit *e*-value was five orders of magnitude higher than the second-best hit *e*-value (Brelsford *et al.* 2016a,b). To visualize the synteny between *R. temporaria* and *X. tropicalis*, we used the software Circos plot v 0.69-2 (Krzywinski *et al.* 2009) as in Yang *et al.* (2014).

### Data availability

File S1 contains a detailed protocol of the library preparation. File S2 contains the pipeline for the construction of the draft genome. File S3 contains detailed information about the microsatellites used (*i.e.*, accession numbers, core motifs, and references). File S4 contains sex-specific and average maps and a comparison with previous microsatellite maps. File S5 contains supplementary figures and tables being: Figure S1, linear model that fits the relation between male and female number of markers; Figure S2, linkage group specific male *vs.* female recombination lengths; Figure S3, map length and number of markers compared to Swiss map; Figure S4, kernel smoothing results; Table S1, summary of distorted maps, linkage group lengths, number of markers, and sex-specific recombination rates. File S6 contains a figure of the distorted sex-specific map. File S7 contains sex-specific distorted maps and the results of the kernel analysis. File S8 contains the genotypic information of the individuals. The draft genome is available at https://figshare.com/s/c0ff6bacbfc4572e1ff1 and marker sequences are available at https://figshare.com/s/24fa6c7cd2f133467207.

## Results

### Microsatellite and RAD analysis

Out of the 113 amplified microsatellites, 77 were heterozygous in at least one parent. Twelve of them possessed null alleles and four loci showed Mendelian segregation violation (*p*-value < 0.0001), resulting in a final set of 61 informative microsatellites (File S3). In the whole dataset, 19 offspring exhibited three alleles in at least one microsatellite locus, suggesting potential partial trisomy, and were excluded from subsequent analysis.

A total of 162 F1 offspring were used to construct the linkage map, after discarding three individuals with < 5000 reads. Approximately 634 million reads were retained after the quality filtering. Over 10 million reads were obtained from each parent, while on average 3.8 million of reads were sequenced per offspring (ranging from 2 to 6.8 million). The assembly from the parental read alignment resulted in 88,497 contigs. From those, 13,203 contigs were polymorphic and were used for mapping the reads from the progeny and SNP calling. Over 13,600 SNPs were initially identified, from which 7217 markers passed a *X^2^* test for Mendelian segregation (*p*-value > 0.05). The estimated genotyping error inferred based on independent technical replicates was ∼5% (5.5% sire and 5.3% dam).

**Figure 1:**
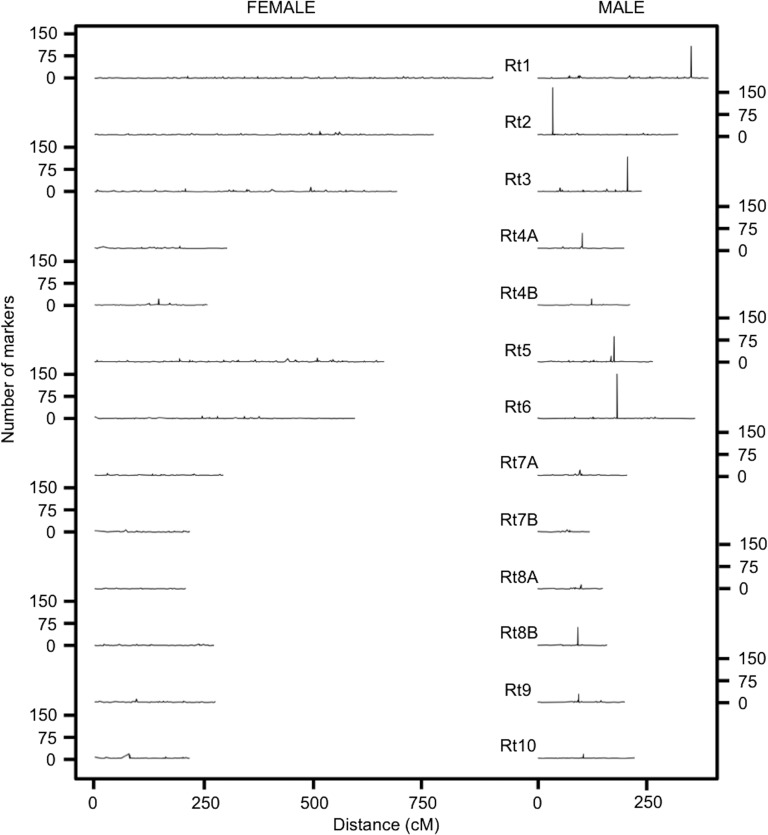
Difference in recombination between sexes. While the female map shows a more uniform recombination rate across all linkage groups, the male map exhibits nonrecombining regions as peaks of high marker density.

Sex-specific linkage maps were constructed using 3644 markers (51 microsatellites and 3593 SNPs) and 3180 markers (51 microsatellites and 3129 SNPs), for female and male respectively. After discarding linkage groups formed by <4 markers, 13 linkage groups were recovered in both maps (Table 1). A total of 3596 markers in the female map and 3105 markers in the male map were assigned to the 13 linkage groups (File S4). Our map presented an observed genome coverage of 99% for both sexes. The average recombination rate was 1.35 times higher and the total length was roughly twofold (1.87 times) larger in the female map than in the male map. Interestingly, all male linkage groups contained a cluster of markers with zero recombination, henceforth referred to as recombination cold spot (Figure 1). The number of clustered nonrecombining markers was higher in the largest linkage groups (*i.e.*, Rt1, Rt2, Rt3, Rt5, and Rt6) ranging from 86 to 159 markers, while 9–62 nonrecombining markers were detected in smaller linkage groups in the male map. The nonrecombining regions in the female map were generally smaller (<23 markers). However, the linkage groups Rt4B, Rt7B, and Rt10 showed nonrecombining regions of similar length in both sexes (Table 1).

**Table 1:** Linkage group length, total number of markers, recombination rate, and number of markers in the nonrecombining region by sex

LG	Length (cM)		No. Markers		Recombination Rate		No. Markers Within Cold Spot	
	Male	Female	Male	Female	Male	Female	Male	Female
Rt1	392.838	914.948	551	631	0.71	1.45	108	8
Rt2	323.074	778.775	435	497	0.74	1.57	159	13
Rt3	239.262	694.948	383	453	0.62	1.53	117	16
Rt4A	199.211	304.537	178	160	1.12	1.9	52	9
Rt4B	212.494	259.109	114	161	1.86	1.61	22	22
Rt5	265.076	664.933	323	481	0.82	1.38	86	7
Rt6	362.237	597.954	422	339	0.86	1.76	150	10
Rt7A	205.762	295.579	144	177	1.43	1.67	19	8
Rt7B	119.879	218.716	70	125	1.71	1.75	9	9
Rt8A	149.881	209.416	85	96	1.76	2.18	15	4
Rt8B	159.626	273.802	135	158	1.18	1.73	62	6
Rt9	200.646	277.64	133	173	1.51	1.6	28	12
Rt10	222.21	217.664	132	145	1.68	1.5	16	17
Total	3052.196	5708.021	3105	3596	1.23	1.67		

LG, linkage group; No., number.

**Figure 2:**
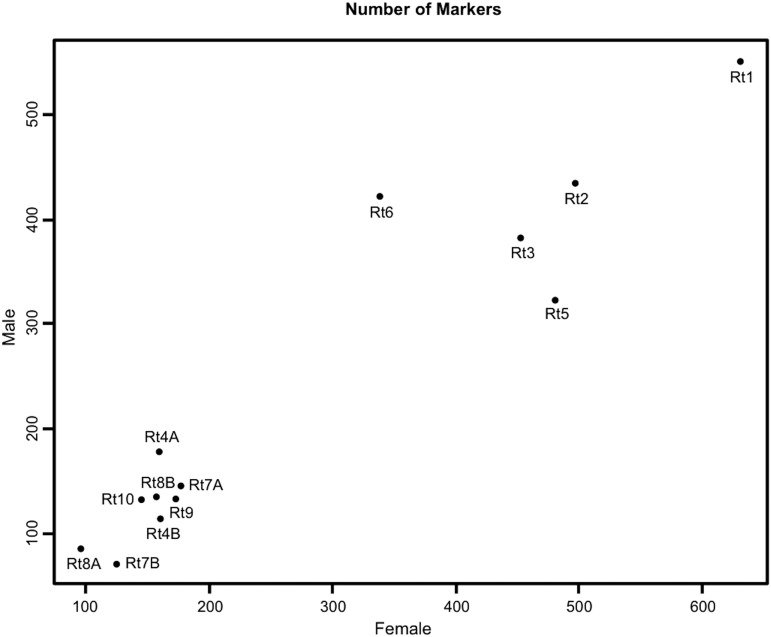
Female *vs.* male number of markers for each linkage group.

Our results showed that the linkage group Rt1 is the longest and contains the largest number of markers in both sexes (Figure 1 and Figure 2). The female map presented a slightly higher number of markers per linkage group, except for -Rt4A and Rt6 (Table 1). Rt6 deviated from the general trend due to a larger number of markers in the male than in the female map (Figure 2 and Figure S1). Furthermore, female map showed higher recombination length than the male map but some linkage groups deviated above or below from the general trend (Figure S2). For instance, Rt6 was longer in the male map and Rt10 was shorter in the female map compared to other linkage groups. Rt3 exhibited the lowest recombination rate in the male map. Finally, Rt7B was the shortest linkage group in the male map with the smallest number of markers (Table 1).

Compared with the recent RAD-based linkage map in the common frog (Brelsford *et al.* 2016b), the number of markers and recombination length of the linkage groups in our study was similar (coefficient of determination, female map length *R^2^* = 0.83, number of markers in female map *R^2^* = 0.92, number of markers in male map *R^2^* = 0.90, all *p*-values < 0.05) except for the length of the male linkage groups (coefficient of determination, *R^2^* = 0.09, *p*-value > 0.05) (Figure S3). The lack of correlation between length of male linkage groups and the number of markers supports the independence between number of crossovers and chromosome size in males.

### Transmission ratio distortion

**Figure 3:**
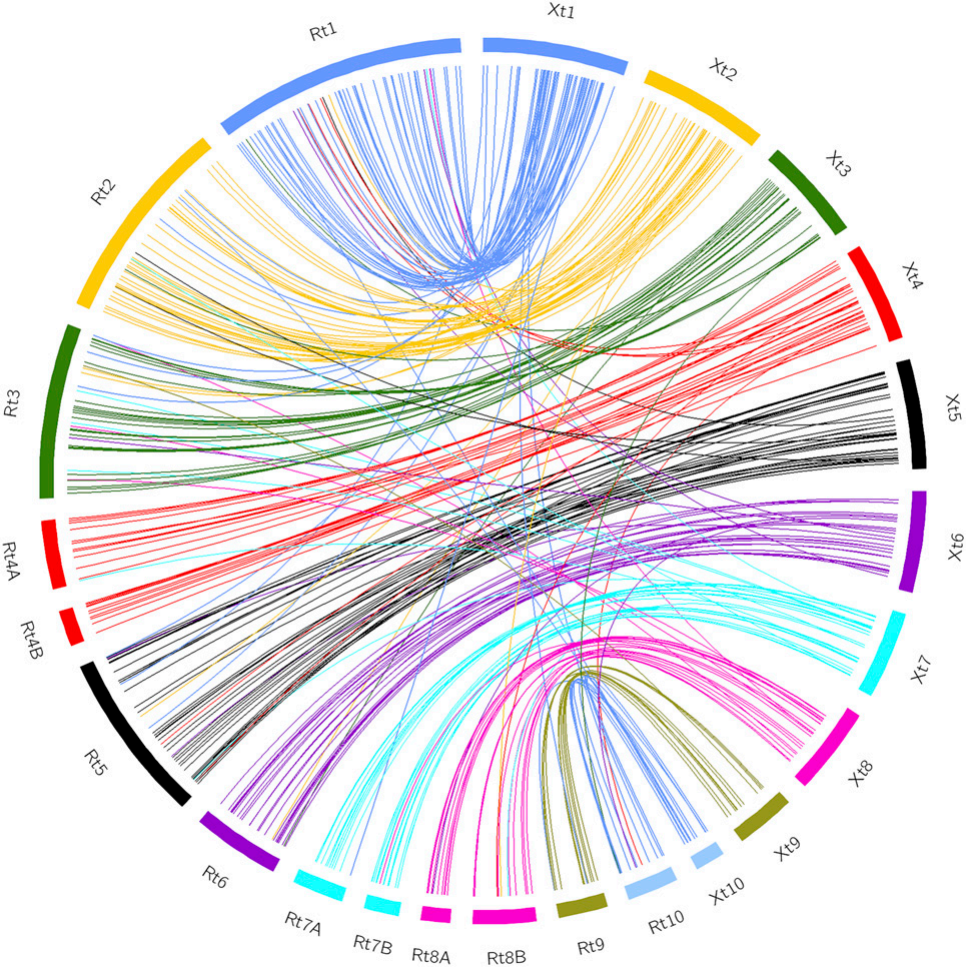
Circos plot showing the strong synteny between *X. tropicalis* and *R. temporaria*. To obtain better visualization, the number of base pairs of the *R. temporaria* linkage groups was multiplied by 100.

In order to evaluate the segregation distortion patterns along the chromosomes, 521 and 401 distorted markers of dam and sire, respectively, were added to construct new sex-specific maps. Among the distorted markers, a total of 500 SNPs segregating in the dam (12.2% of total number of markers) and 375 SNPs segregating in the sire (10.8% of total number of markers) were successfully assigned within the 13 linkage groups (File S7). The resulting map was 663 cM longer in the female and 398 cM longer in the male compared to the maps without including distorted markers. Average recombination rate was similar in distorted and nondistorted maps (Table S1). The distribution of the distorted markers exhibited a nonuniform pattern along and among linkage groups (File S6). For example, a large number of distorted markers was observed on Rt1 in contrast with the low number on Rt4B and Rt7A. Furthermore, there were differences between sexes. Kernel smoothing results showed a higher distortion in the female than in the male map (Figure S4). Only Rt2, Rt5, Rt7B, Rt8B, and Rt9 from the male map exhibited regions with signals of high distortion based on the kernel analysis, while all female linkage groups except Rt9 contained at least one area with an excess of distorted markers. Interestingly, Rt6 and Rt9 from the male map and Rt7B from the female map showed clusters of distorted markers within the recombination cold spots (Figure 3).

### Average map

We used 7278 markers (61 microsatellites and 7217 SNPs) to construct the average linkage map and 7138 markers were successfully assigned to 13 linkage groups. All the linkage groups reached stabilization after five Monte Carlo Expectation Maximization cycles. All markers were located within the same linkage groups as in sex-separated maps. However, as expected, the average map showed less accurate marker order than the female map.

### Similarity analysis

A total of 4161 forward and reverse reference sequences from the sire were aligned to the *R. temporaria* draft genome. In 2359 cases (56.7%), the pair of forward and reverse reference sequences aligned onto the same scaffold within 600 bp (range from 190 to 473 bp). From a total of 2331 scaffolds selected, 1184 aligned to the *N. parkeri* genome (50.8%) and 305 aligned to the *X. tropicalis* genome (13%). In addition, 81% of homologous sequences between *R. temporaria* and *X. tropicalis* were located in the same chromosome and the synteny was also strong, as is shown in the Circos plot (Figure 3).

## Discussion

We have generated the densest linkage map for the common frog to date. The combined use of SNPs and microsatellites allowed in-depth comparison with the existing linkage maps for the species allowing, for the first time, a detailed analysis of the early diverged Iberian lineages in relation to the more recently diverged Western and Eastern lineages.

Both the number and relative length of our linkage groups matched the karyotype described for the species, with five large and eight short chromosomes (Spasić-Bošković *et al.* 1997). Furthermore, we observed a strong synteny between the *R. temporaria* linkage map and the genome of *X. tropicalis*. Heterochiasmy was pronounced, with a lower recombination rate in the male map. Finally, some regions exhibited a strong transmission ratio distortion, which might be indicative of genetic incompatibilities between the lineages used in our experimental cross. Below, we discuss in detail the recombination patterns and evolutionary processes that may have shaped the differences found among the lineages of *R. temporaria*.

### Comparison with previous microsatellite maps

The obtained consensus linkage map was consistent with previous microsatellite maps based on the Western and Eastern lineages (Cano *et al.* 2011; Rodrigues *et al.* 2013, 2016). However, compared to the first linkage map based on Swedish populations from the Eastern lineage (Cano *et al.* 2011), our map revealed several differences. For example, markers that showed significant linkage in groups 1, 4, and 12 from Cano *et al.* (2011) were unlinked in our map and located in other linkage groups (File S4). On the other hand, linkage groups 15 and 2 from Cano *et al.* (2011) were joined within the largest group, Rt1, in our map (File S4). Separation of these two linkage groups in Cano *et al.* (2011) was likely due to lower coverage. Since Rodrigues *et al.* (2016) also found similar inconsistencies in their map based on Swedish populations, the observed discrepancies may be caused by differences in coverage and methodology. Nevertheless, our results revealed frequent change in marker order compared to the published maps (File S4), hence, further research is needed to determine if some of these changes can reflect the occurrence of independent chromosomal rearrangements, such as inversions, among the different lineages.

### Recombination rate

Similar to earlier studies, we detected large differences in recombination rate between sexes (Berset-Brändli *et al.* 2008; Cano *et al.* 2011; Rodrigues *et al.* 2013; Brelsford *et al.* 2016b). The average recombination rate was 1.36 times larger in the female than in the male map. This heterochiasmy is smaller than that observed in maps from the Eastern (1.76 times; Cano *et al.* 2011) and Western lineages, both based solely on microsatellite markers (82.5 times; Rodrigues *et al.* 2013) and SNPs (3.15 times; Brelsford *et al.* 2016b). In relation to the total length of the maps, our female map was 1.87 times larger than the male map, similar to the first Eastern clade map (1.52 times, Cano *et al.* 2011) but more discordant with the second Eastern clade map (17.4 times, Rodrigues *et al.* 2016) and the Western clade maps, which ranged from 3.37 to 72 times (Rodrigues *et al.* 2013; Brelsford *et al.* 2016b). All in all, the heterochiasmy pattern observed in this study was similar to the first Eastern lineage map (Cano *et al.* 2011), which was also based on an outcross. More families from the North Iberian lineages would be needed to confirm these findings.

Concerning the sex-specific recombination patterns, female recombination rates were fairly constant overall while male recombination rates were reduced in the majority of the linkage groups (Figure 1). Similar to Brelsford *et al.* (2016b), a cluster of markers with no recombination occurred in every chromosome, forming an extensive suppressed-recombination region in the largest linkage groups. In amphibians, male recombination is usually restricted to telomeric areas and the nonrecombining region is near the center of the linkage group (Morescalchi and Galgano 1973), coinciding with the centromere and paracentromere area (King 1991). This pattern was found in *Hyla arborea* (Brelsford *et al.* 2016a), and it is in accordance with the metacentric/submetacentric chromosomes described in the karyotype of *R. temporaria* based on populations from the Eastern lineage (Spasić-Bošković *et al.* 1997). However, the position of the nonrecombining regions in our map was shifted to one side in Rt1, Rt2, and Rt3 (Figure 1). Furthermore, subtelomeric/telomeric recombination cold spots were also observed in some linkage groups of the Western clade [see Figure 1 in Brelsford *et al.* (2016b)]. The authors suggested that the shifted position in their map could be due to a lack of coverage at the chromosome ends (A. Brelsford, personal communication). However, because of the higher coverage of the current map and the good homology with the terminal regions of the chromosomes in *X. tropicalis*, the map presented here reflects the actual position of the recombination cold spots in the chromosomes well. Hence, our results call for further karyotype and whole-genome sequencing work between the Iberian and more recently diverged lineages to establish whether centromere repositioning has occurred in the species.

The Iberian Peninsula has been suggested as the place where *R. temporaria* originated (Vences *et al.* 2013) and the Northwest Iberian lineages diverged 1.12 MYA from the lineage for which the karyotype of the species was described (*i.e.*, Balkans), being isolated in different refugia during the last glaciation. Centromere repositioning events have been observed at similar evolutionary time scales but only at the interspecific level. For example, five centromere repositioning events have occurred between donkeys and zebras, which diverged from each other 1–2.78 MYA (Carbone *et al.* 2006; Vilstrup *et al.* 2013).

### Putative sex linkage group

*R. temporaria*, as is the case in other *Rana* species, exhibits male heterogamety (XY). If sex determination is strictly genetic and there is no recombination between sex-chromosomes, X and Y are expected to accumulate differences as a result of large rearrangements and/or degeneration of the Y chromosome (Charlesworth and Charlesworth 2000; Charlesworth *et al.* 2005). Thus, over time, heterogamety is expected to result in an excess of heterozygotes in the linkage group(s) containing the sex-determining gene(s). Based on this expectation, and evidence in *H. arborea* frogs, Brelsford *et al.* (2016a) proposed a method to detect sex chromosomes based solely on heterozygosity differences among linkage groups.

Nevertheless, X and Y chromosomes are homomorphic in the common frog and recombination between them still seems to occur as reported for other amphibian species (Stöck *et al.* 2013; Dufresnes *et al.* 2014). Perrin (2009) suggested that occasional sex-reversal events could maintain X–Y recombination because XY females would prevent the decay of the Y chromosome. In fact, spontaneous sex-reversed individuals, including observed XX males and possibly XY females, have been documented in some populations (Matsuba *et al.* 2008; Perrin 2009; Alho *et al.* 2010). Previous studies found several sex-linked markers in the linkage group Rt1 and it has been considered as the putative sex chromosome for the species (*e.g.*, Matsuba *et al.* 2008; Alho *et al.* 2010; Cano *et al.* 2011; Rodrigues *et al.* 2013). However, both Brelsford *et al.* (2016b) and our study failed to find the expected heterozygosity excess in Rt1 as a putative sex chromosome. Since we lacked phenotypic sex information for our family, we cannot establish whether nongenetic sex determination plays a role in our results, although this was the case in Brelsford *et al.* (2016b).

On the other hand, Rt6 showed markedly higher male heterozygosity in our cross (Figure 2 and Figure S1) and harbored two sex-linked markers in the Finnish population of Kilpisjärvi (BFG267 and BFG239; C. Matsuba, unpublished data). Therefore, this linkage group warrants further research to determine its potential involvement in sex determination or whether another source of differentiation among the lineages crossed is causing this large male heterozygosity. The possibility of multiple chromosomes participating in the sex determination of the common frog has also been discussed in previous work (Cano *et al.* 2011; Rodrigues *et al.* 2013, 2014) and demonstrated recently by Rodrigues *et al.* (2016). Alternatively, a large autosomal supergene with two differentiated haplogroups in the crossed lineages could also produce higher male heterozygosity, if the male were heterozygous and the female were homozygous (Brelsford *et al.* 2016a).

### Segregation distortion

Many biological processes acting before or after fertilization can cause transmission ratio distortion (Fishman *et al.* 2001; Kuittinen *et al.* 2004). We found clusters of distorted markers in the nonrecombining regions of the linkage groups Rt6 and Rt9 in the male and Rt7B in the female, based on the randomization and kernel smoothing approach. There are nongenetic sources of segregation distortion such as genotyping errors, sampling biases, and comigration (Rogers *et al.* 2007; Zhou *et al.* 2015). Our genotyping-by-sequencing dataset contained ∼5% of genotyping errors based on analysis of replicated samples, despite the rather strict quality control. However, such technical artifacts are not expected to systematically cluster together in relation to other loci. Thus, the distorted genomic regions with blocks of distorted markers found in this study are likely related to biological processes such as meiotic drive, lineage incompatibilities, or outbreeding depression (Zhou *et al.* 2015). Since we observed high mortality and deformity rates in our experiment, these genomic regions are good candidates to investigate potential genetic incompatibilities. Furthermore, loci with non-Mendelian inheritance could have greater evolutionary importance than our current knowledge suggests (Lyttle 1991; Taylor and Ingvarsson 2003). However, analysis of gametes and replicate families generated preferably using backcross breeding design would be needed to further understand the relationship between genome structure and segregation distortion.

### Homology analysis

The comparative genomic analysis between *R. temporaria*, *X. tropicalis*, and *N. parkeri* supported the strong homology among amphibian genomes (Brelsford *et al.* 2013, 2016a). As expected from their close phylogenetic relationship (Pyron and Wiens 2011), *N. parkeri* showed higher homology with *R. temporaria*. These species diverged 90 MYA while *X. tropicalis* and *R. temporaria* split ∼208 MYA (divergence times retrieved from timetree.org). The estimated homology between *R. temporaria* and *X. tropicalis* in this study (12.8%) is slightly higher than that observed (10%) for the Western lineage of this species (Brelsford *et al.* 2016b). Our study confirmed the finding of Brelsford *et al.* (2016b), that *X. tropicalis* chromosomes 4, 7, and 8 were split into two pairs in *R. temporaria*.

### Conclusions

The constructed high-density consensus linkage map provides an important resource for further research in the evolutionary biology of *R. temporaria*, facilitating the search for genes of adaptive relevance. In addition, due to the conserved synteny among amphibians, this linkage map represents a valuable tool for further comparative genomic studies. Our work indicates that the genome structure is generally conserved between common frog lineages while the position of the recombination cold spots and marker order can vary. Finally, genomic regions showing strong transmission distortion found here are promising candidates for studying incipient speciation processes.

## Supplementary Material

Supplemental material is available online at www.g3journal.org/lookup/suppl/doi:10.1534/g3.116.036459/-/DC1.
