# Supplementary material for: Comparative High-Density Linkage Mapping Reveals Conserved Genome Structure but Variation in Levels of Heterochiasmy and Location of Recombination Cold Spots in the Common Frog
Source: G3 (Bethesda). 2016 Dec 28;7(2):637–45. doi: 10.1534/g3.116.036459 (PMC5295608; doi:10.1534/g3.116.036459)
Supplement: Supplementary file 1 [file 637FigureS1.docx]

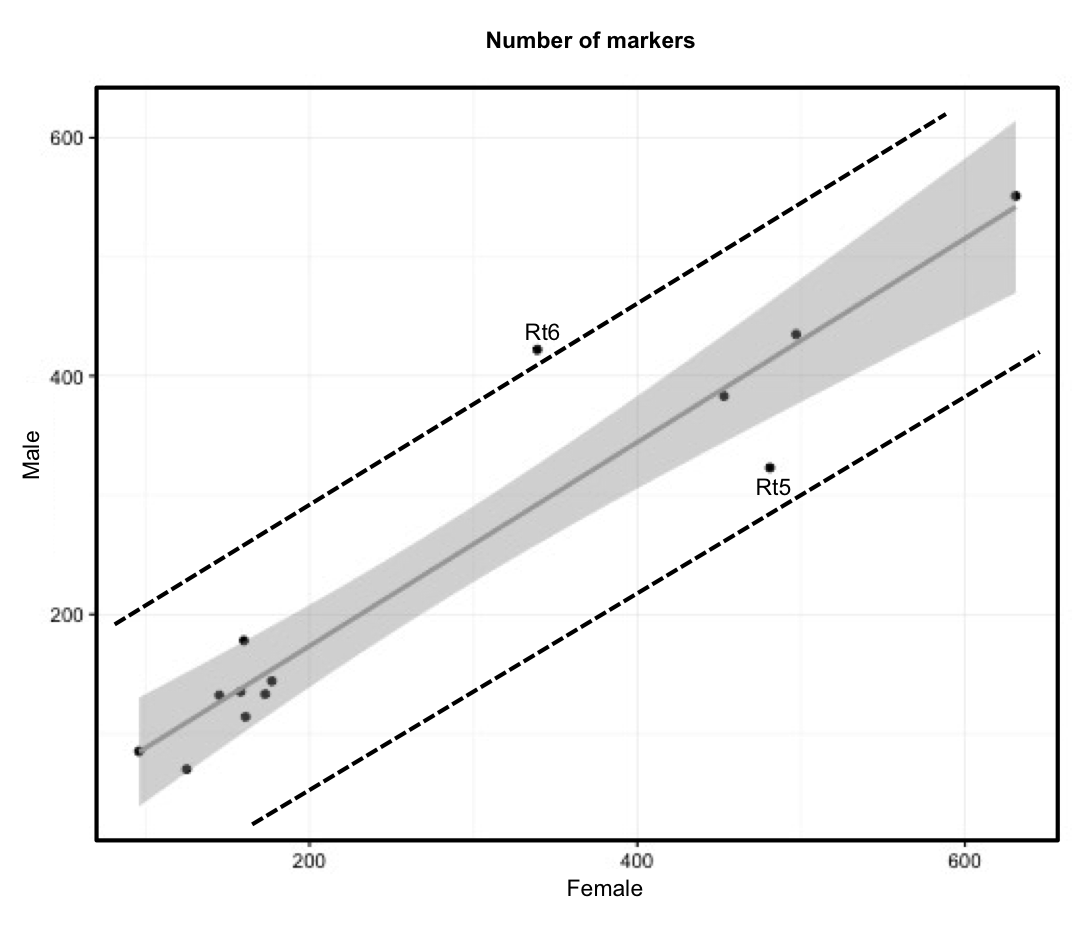


**Fig. S1**: Female vs. male number of markers for each linkage group. Grey line defines the linear regression, grey area represents the 95% confident intervals and dashed lines delimit the predicted area of the linear model.
