# Supplementary figures and images for: Comparative High-Density Linkage Mapping Reveals Conserved Genome Structure but Variation in Levels of Heterochiasmy and Location of Recombination Cold Spots in the Common Frog
Source: G3 (Bethesda). 2016 Dec 28;7(2):637–45. doi: 10.1534/g3.116.036459 (PMC5295608; doi:10.1534/g3.116.036459)

**Fig. S2**: Female vs. male recombination length for each linkage group.


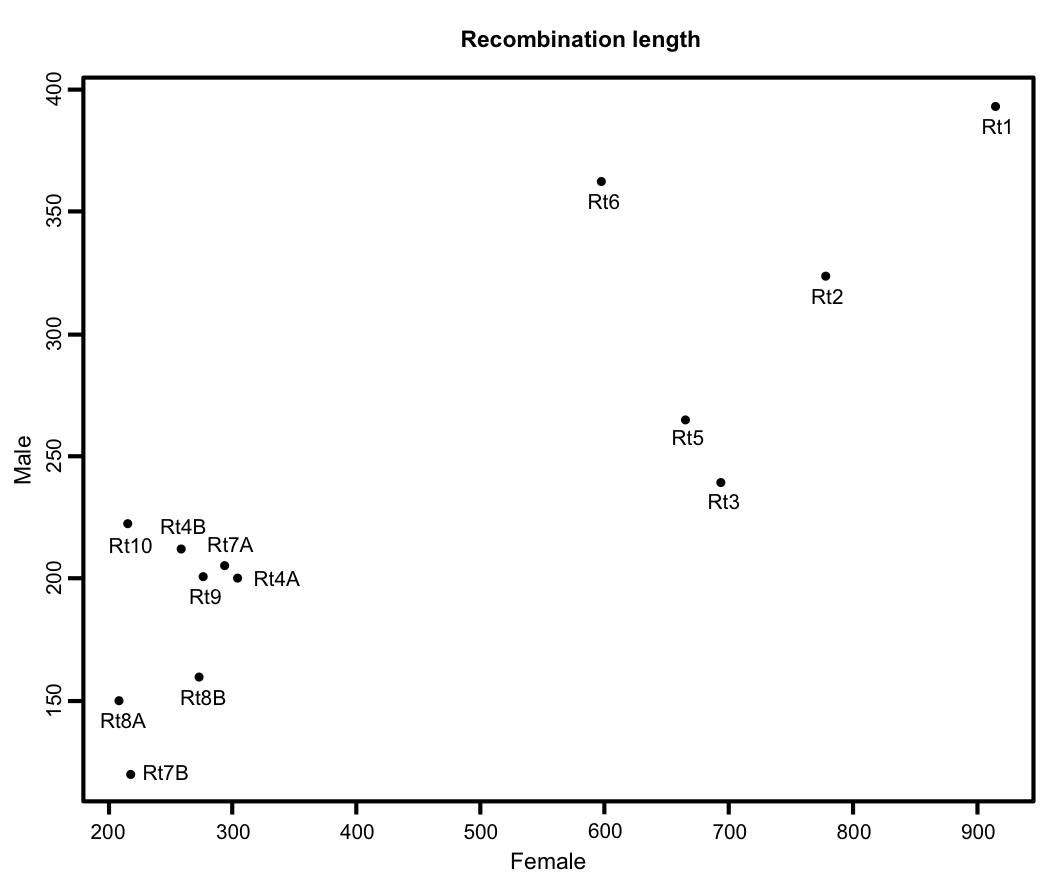

Supplement: Supplementary file 2 [file 637FigureS2.docx]

**Fig. S3**: Comparison between maps from Spanish and Swiss populations.


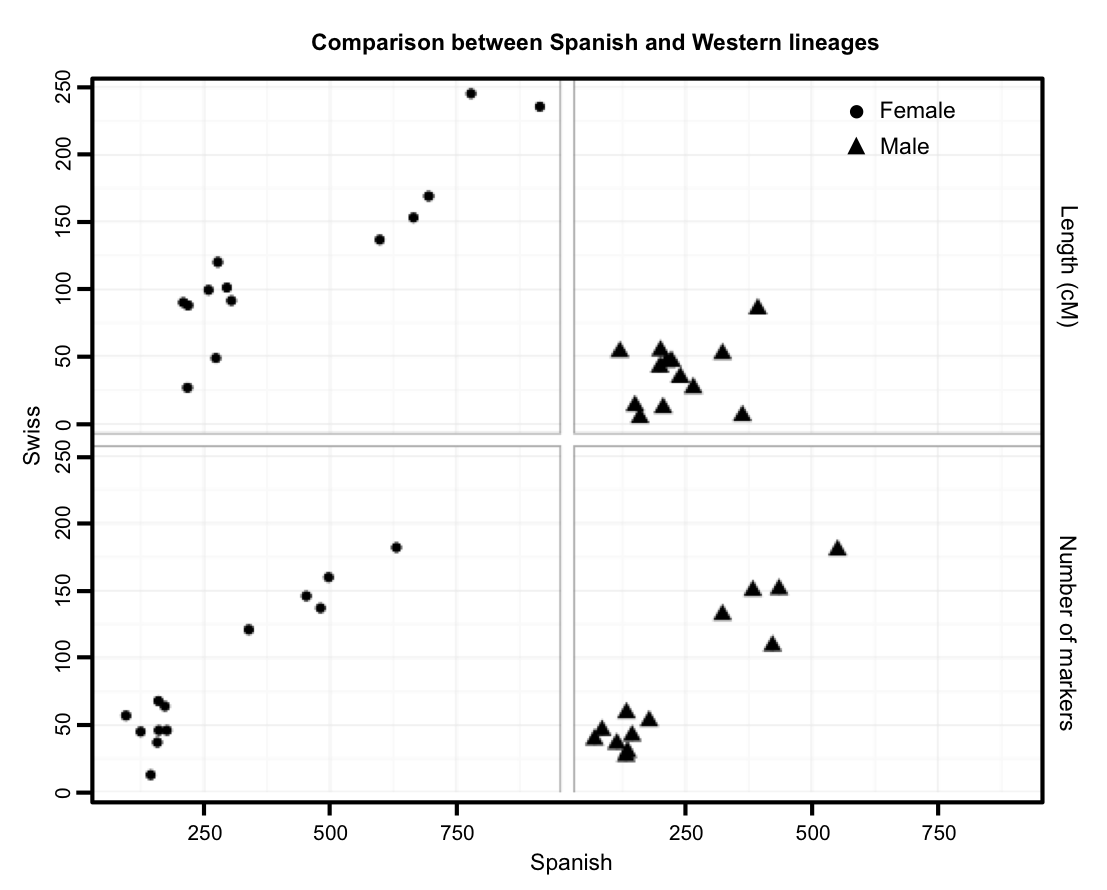

Supplement: Supplementary file 3 [file 637FigureS3.docx]

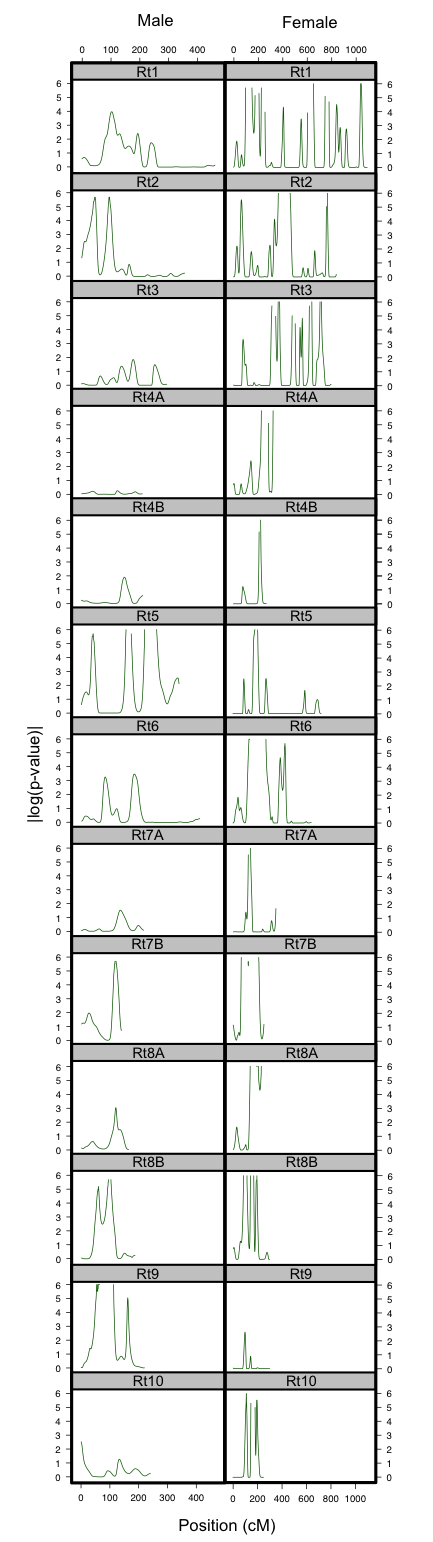
**Fig. S4**: Results of kernel smoothing analysis.

Supplement: Supplementary file 4 [file 637FigureS4.docx]
