# Supplementary material for: Comparative High-Density Linkage Mapping Reveals Conserved Genome Structure but Variation in Levels of Heterochiasmy and Location of Recombination Cold Spots in the Common Frog
Source: G3 (Bethesda). 2016 Dec 28;7(2):637–45. doi: 10.1534/g3.116.036459 (PMC5295608; doi:10.1534/g3.116.036459)
Supplement: Supplementary file 5 [file 637TableS1.docx]

| **LG** | **Length (cM)** | | **Number of markers** | | **Number of distorted markers** | | **Recombination rate** | |
| --- | --- | --- | --- | --- | --- | --- | --- | --- |
|  |  |  |  |  |  |  |  |  |
|  | **Male** | **Female** | **Male** | **Female** | **Male** | **Female** | **Male** | **Female** |
| **Rt1** | 461.529 | 1092.235 | 600 | 729 | 49 | 98 | 0.77 | 1.5 |
| **Rt2** | 357.213 | 840.877 | 479 | 569 | 44 | 72 | 0.75 | 1.48 |
| **Rt3** | 296.346 | 798.863 | 412 | 497 | 29 | 44 | 0.72 | 1.61 |
| **Rt4A** | 210.451 | 334.516 | 190 | 202 | 12 | 42 | 1.11 | 1.66 |
| **Rt4B** | 212.768 | 268.835 | 121 | 170 | 7 | 9 | 1.76 | 1.58 |
| **Rt5** | 338.977 | 720.773 | 397 | 516 | 74 | 35 | 0.85 | 1.4 |
| **Rt6** | 410.171 | 639.857 | 447 | 397 | 25 | 58 | 0.92 | 1.61 |
| **Rt7A** | 215.525 | 348.734 | 151 | 197 | 7 | 20 | 1.43 | 1.77 |
| **Rt7B** | 138.085 | 252.042 | 88 | 160 | 18 | 35 | 1.57 | 1.58 |
| **Rt8A** | 163.389 | 231.488 | 100 | 120 | 15 | 24 | 1.63 | 1.93 |
| **Rt8B** | 185.5 | 295.147 | 165 | 182 | 30 | 24 | 1.12 | 1.62 |
| **Rt9** | 219.621 | 297.602 | 187 | 187 | 54 | 14 | 1.17 | 1.59 |
| **Rt10** | 240.479 | 250.128 | 143 | 170 | 11 | 25 | 1.68 | 1.47 |
| **TOT** | **3450.054** | **6371.097** | **3480** | **4096** | **375** | **500** | **1.19** | **1.6** |
