# Supplementary material for: Comparative High-Density Linkage Mapping Reveals Conserved Genome Structure but Variation in Levels of Heterochiasmy and Location of Recombination Cold Spots in the Common Frog
Source: G3 (Bethesda). 2016 Dec 28;7(2):637–45. doi: 10.1534/g3.116.036459 (PMC5295608; doi:10.1534/g3.116.036459)
Supplement: Supplementary file 6 [file 637FileS1.docx]

Appendix 1

**Adapter preparation**

Oligonucleotides (i.e. top and bottom strand of the adapter) were annealed to create the double-stranded adapters. Their initial concentration was 250 μM. We prepared solutions 50 μM mixing 20 μl of top strand, 20 μl of bottom strand and 60 μl of TE. This mix was heated at 100°C in a water bath for 5 minutes and subsequently cooled slowly until room temperature.

Two different types of adapters were prepared:

🡪 “Barcode” adapter

5′-ACACTCTTTCCCTACACGACGCTCTTCCGATCTxxxx**TGCA**

5′-yyyyAGATCGGAAGAGCGTCGTGTAGGGAAAGAG​TGT

These adapters include 4 to 8 bp barcode (xxxx and its complementary yyyy). We used following barcodes:

| CTCC | TTCTC | TCGTT | CTATTA | AATATGC | TGCAAGGA |
| --- | --- | --- | --- | --- | --- |
| TGCA | AGCCC | GGTTGT | GCCAGT | ACGTGTT | TGGTACGT |
| ACTA | GTATT | CCAGCT | GGAAGA | ATTAATT | TCTCAGTC |
| CAGA | CTGTA | TTCAGA | GTACTT | ATTGGAT | CCGGATAT |
| AACT | ACCGT | TAGGAA | GTTGAA | CATAAGT | CGCCTTAT |
| GCGT | GCTTA | GCTCTA | TAACGA | CGCTGAT | AACCGAGA |
| CGAT | GGTGT | CCACAA | TGGCTA | CGGTAGA | ACAGGGAA |
| GTAA | AGGAT | CTTCCA | TATTTTT | CTACGGA | ACGTGGTA |
| AGGC | ATTGA | GAGATA | CTTGCTT | GCGGAAT | CCATGGGT |
| GATC | CATCT | ATGCCT | ATGAAAC | TAGCGGA | CGCGGAGA |
| TCAC | CCTAC | AGTGGA | AAAAGTT | TCGAAGA | CGTGTGGT |
| TGCGA | GAGGA | ACCTAA | GAATTCA | TCTGTGA | GCTGTGGA |
| CGCTT | GGAAC | ATATGT | GAACTTC | TGCTGGA | GGATTGGT |
| TCACC | GTCAA | ATCGTA | GGACCTA | ACGACTAC | GTGAGGGT |
| CTAGC | TAATA | CATCGT | GTCGATT | TAGCATGC | TATCGGGA |
| ACAAA | TACAT | CGCGGT | AACGCCT | TAGGCCAT | TTCCTGGA |

🡪 “Common” adapter

5′-**GATC**AGATCGGAAGAGCGGTTCAGCAGGAATGCCGAG

5′-CTCGGCATTCCTGCTGAACCGCTCTTCCGATCT

Both types of adapters present 4bp that are complementary to the “sticky” ends of the restriction enzymes. Barcode adapter terminates with TGCA on 3’ end of its top strand which fits with PstI cut. Common adapter starts with GATC on 5’end of its top strand that matches with BamHI cut.

After the annealing, each adapter was quantified with Qubit® fluorometer and diluted to 0.5 pmol/μl.

**Digestion**

| **Reagent** | **Volume** |
| --- | --- |
| DNA (100 ng/μl) | 2 μl |
| dH_2_O | 15 μl |
| NEBuffer (10X) | 2 μl |
| Enzyme BamHI (20U/μl)* | 0.5 μl |
| Enzyme PstI (20U/μl)* | 0.5 μl |

* Restriction enzymes from New England BioLabs

Samples were digested for 2 h at 37°C, followed by a heat-inactivation of the enzymes for 15 min at 75°C.

**Ligation**

| **Reagent** | **Volume** |
| --- | --- |
| Digestion product | 20 μl |
| dH_2_O | 20 μl |
| Barcode adapter (0.5 pmol/μl)* | 1 μl |
| Common adapter (0.5 pmol/μl)* | 1 μl |
| Buffer T4 Ligase (10X) | 5 μl |
| T4 Ligase (400 U/μl)** | 3 μl |

* Adapters from Life Technologies

** Enzymes from New England BioLabs

Samples were ligated for 1h at 22°C followed by the heat-inactivation for 30min at 65°C.

After ligation, 10 μl of 94 digested DNA samples, each with different barcode adapter, were combined. A total of 940 μl of ligated DNA per library was obtained.

**Purification**

Purification was carried out with the QIAquick PCR purification kit (QIAGEN) according to the recommended protocol. Samples were eluted in 30 μl of elution buffer.

**E-gel size selection**

E-Gel® iBase™ Power System was used to select fragments from 300 to 600 bp following the instructions from the manufacturer.

**PCR**

| **Reagent** | **Volume** |
| --- | --- |
| Size selected DNA | 15 μl |
| PCR Supermix High Fidelity | 50 μl |
| Primer F (10 μM) | 2 μl |
| Primer R (10 μM) | 2 μl |

Fragments were amplified by a PCR with the following conditions:

20 min 72°C

5 min 95°C

18 cycles of

15 s 95°C

15 s 64°C

30 s 68°C

5 min 72°C

**Primers**

Forward

5′-AATGATACGGCGACCACCGAGATCTACACTCTTTCCC​TACACGACGCTCTTCCGATCT

Reverse

5′-CAAGCAGAAGACGGCATACGAGATCGGTCTCGGCATT​CCTGCTGAACCGCTCTTCCGATCT

**Purification**

After PCR, purification was carried out again with the QIAquick PCR purification kit (QIAGEN) according to the recommended protocol. Samples were eluted in 30 μl of elution buffer.
