# Supplementary material for: Comparative High-Density Linkage Mapping Reveals Conserved Genome Structure but Variation in Levels of Heterochiasmy and Location of Recombination Cold Spots in the Common Frog
Source: G3 (Bethesda). 2016 Dec 28;7(2):637–45. doi: 10.1534/g3.116.036459 (PMC5295608; doi:10.1534/g3.116.036459)
Supplement: Supplementary file 7 [file 637FileS2.docx]

Appendix 2

**Generation of the draft *Rana temporaria* genome**

The individuals (ID: 935 and 931) used for genome sequencing were siblings from a family of Viango population (Spain). Individual DNA was extracted from whole body of tadpoles separately, by using the AllPrep DNA/RNA mini kit (Qiagen). A shotgun library from ID935 DNA was constructed with the TruSeq DNA Sample Prep kit (Illumina, CA). A 3kb Mate-pair (MP) library from ID935 DNA and a 8kbMP library from mixed DNA of ID935 and ID931 were constructed with the Nextera Mate Pair library Sample Prep kit (Illumina, CA). The average fragment size of the three libraries: shotgun, 3kbMP, and 8kbMP, were 500bp, 3.5kb, and 8kb respectively. Libraries were sequenced at seven lanes on Illumina HiSeq2500 sequencer for 100 or 150 cycles. Library construction and sequencing runs were performed at the Roy J. Carver Biotechnology Center in University of Illinois at Urbana-Champaign (Illinois, US). We gained about 2 billion reads corresponding to 322 billion bp in total from three libraries. Considering that shotgun library of 1 billion reads corresponds to 150 billion bases, the estimated coverage of the sequenced genome is around 38X, assuming that the expected genome size of the common frog genome size is 4x10^9^bp. Error correction was performed for the paired end (PE) reads of shotgun sequencing by using the ErrorCorrectReads.pl from ALLPATHS-LG assembler (http://www.broadinstitute.org/software/allpaths-lg/blog/?p=577) . MP reads with at least one pair containing the adaptor were filtered by using the Nextclip (http://www.tgac.ac.uk/nextclip/). The assembly was carried out in three steps using Platanus version 1.2.1 (http://platanus.bio.titech.ac.jp/). Firstly, the cleaned PE reads were assembled with an initial kmer length of k=32 that was automatically increased up to 52. Secondly, Platanus performed the scaffolding step, adding MP reads sequences to the previous assembly and filling some gaps. Finally, Platanus implemented the gap-closing step with a guide of the transcriptome assembly (in preparation). The result of Core eukaryotic genes dataset Analysis (CEGMA) was summarized in the Appendix 2 Table 1. The draft assembly file”frog_3.0.fa.gz” will be available at FigShare.

Table 1. CEGMA results of the draft genome assembly of *Rana temporaria*.

| Number of contigs | 16.3e^6^ |
| --- | --- |
| NG50 | 1180 bp |
| N50 | 1730 bp |
| CEGMA: % of Complete gene sequences | 43.95 |
| CEGMA: % of Partial gene sequences | 89.11 |
