# Supplementary material for: Comparative High-Density Linkage Mapping Reveals Conserved Genome Structure but Variation in Levels of Heterochiasmy and Location of Recombination Cold Spots in the Common Frog
Source: G3 (Bethesda). 2016 Dec 28;7(2):637–45. doi: 10.1534/g3.116.036459 (PMC5295608; doi:10.1534/g3.116.036459)
Supplement: Supplementary file 10 [file 637FileS4.docx]

File S4. Sex specific and average maps and a comparison with previous microsatellite maps. (.xlsx, 509 KB)

[http://www.g3journal.org/lookup/suppl/doi:10.1534/g3.116.036459/-/DC1/FileS4.xlsx](http://www.g3journal.org/lookup/suppl/doi:10.1534/g3.116.036459/-/DC1/FileS3.xlsx)
