# Supplementary material for: Comparative High-Density Linkage Mapping Reveals Conserved Genome Structure but Variation in Levels of Heterochiasmy and Location of Recombination Cold Spots in the Common Frog
Source: G3 (Bethesda). 2016 Dec 28;7(2):637–45. doi: 10.1534/g3.116.036459 (PMC5295608; doi:10.1534/g3.116.036459)

Distorted map. Each figure represents a linkage group (left male and right female). Markers shown on the right side are those deviated from Mendelian segregation. Their position in the linkage group is shown on the left side. Non-distorted markers are presented as tight bands in the linkage group.


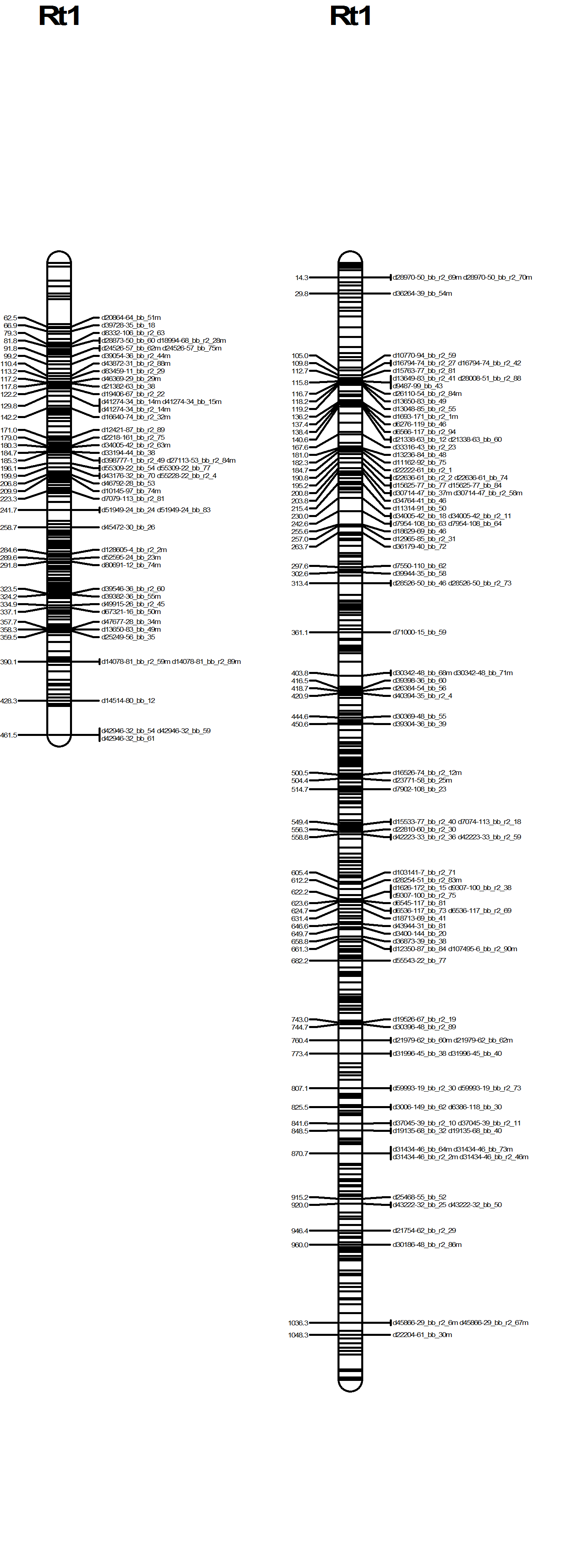

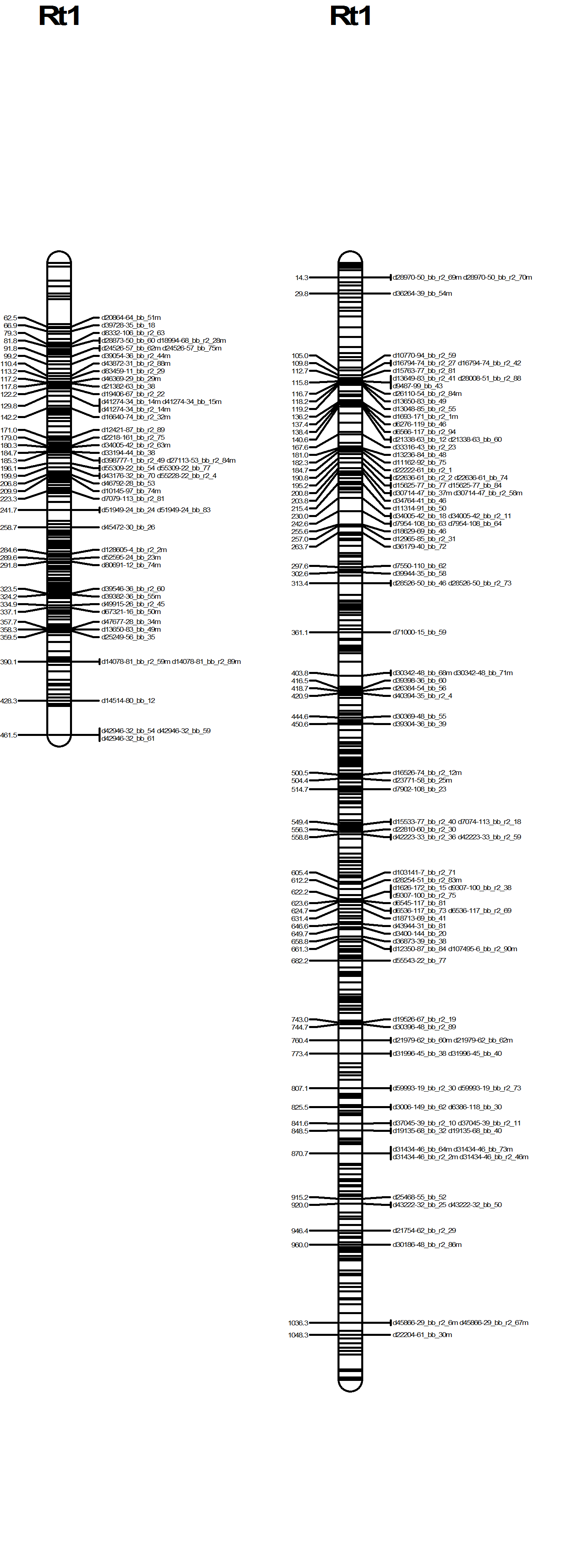


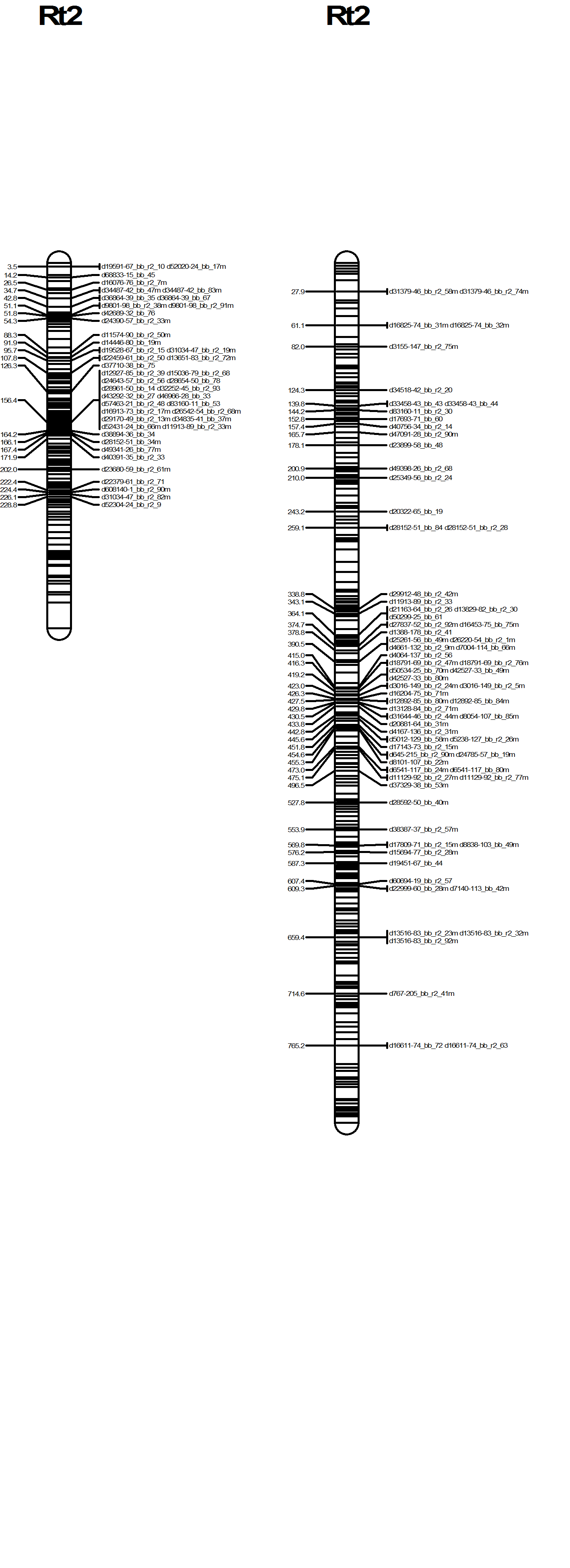


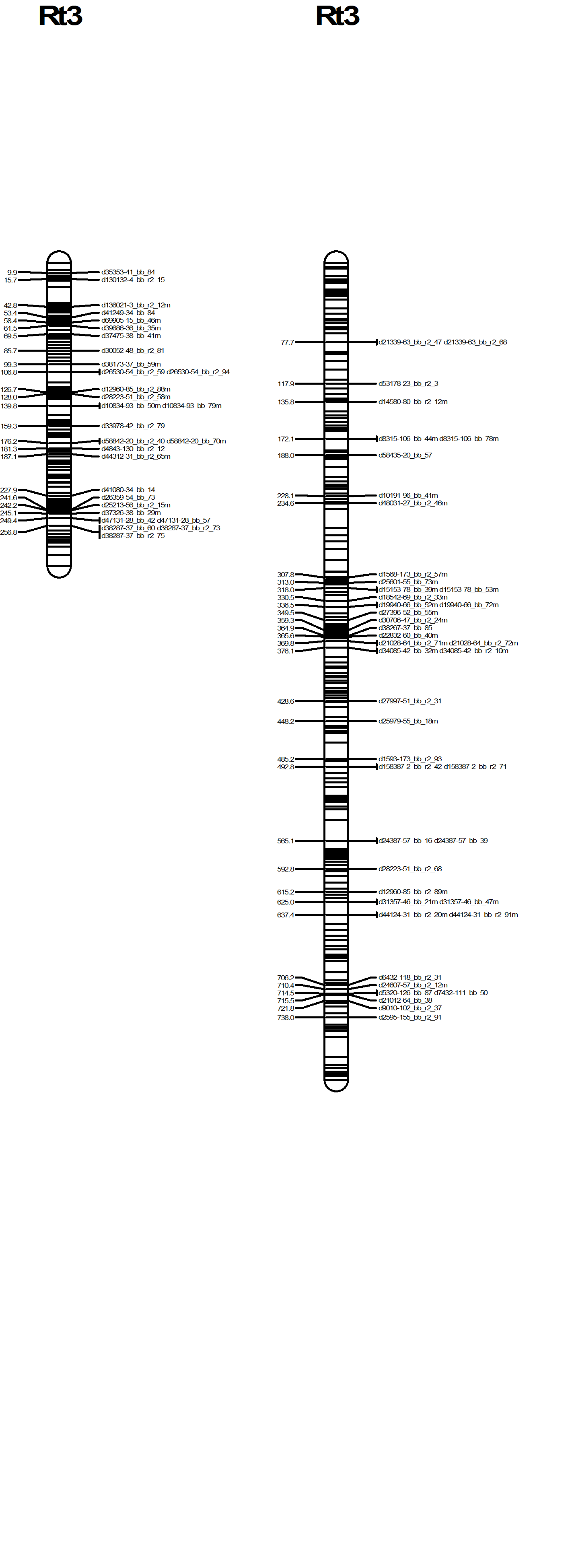


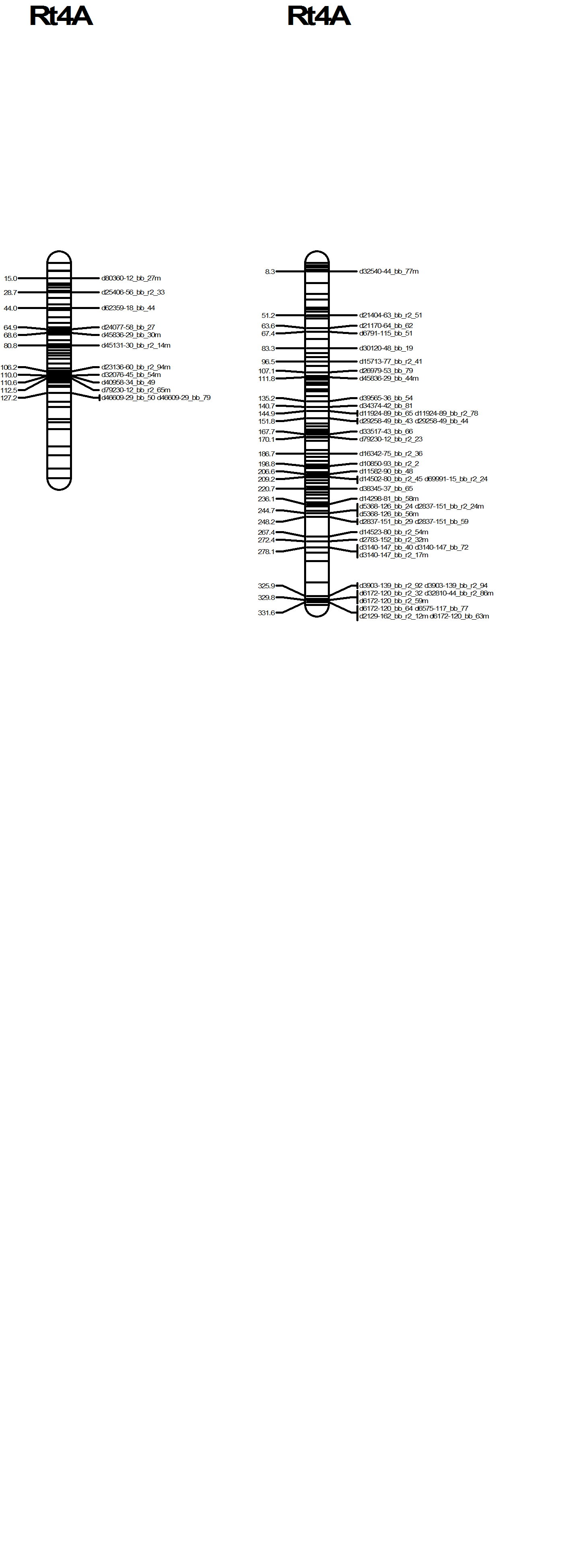


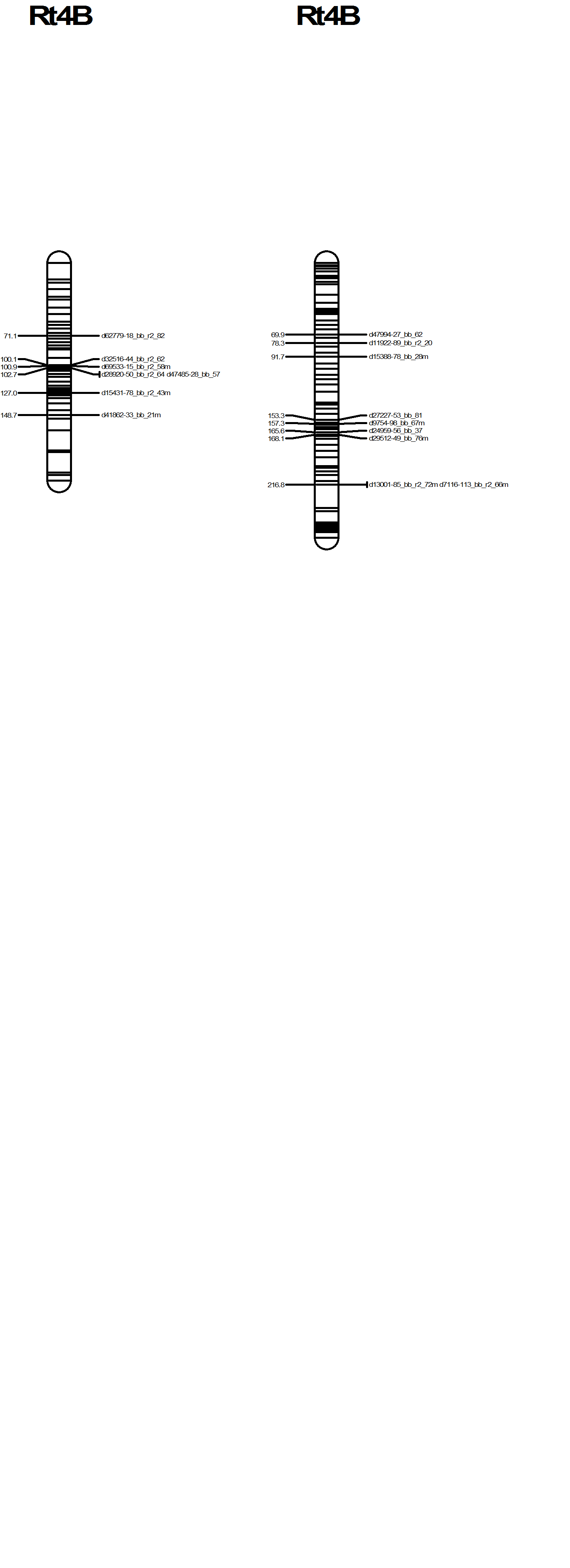


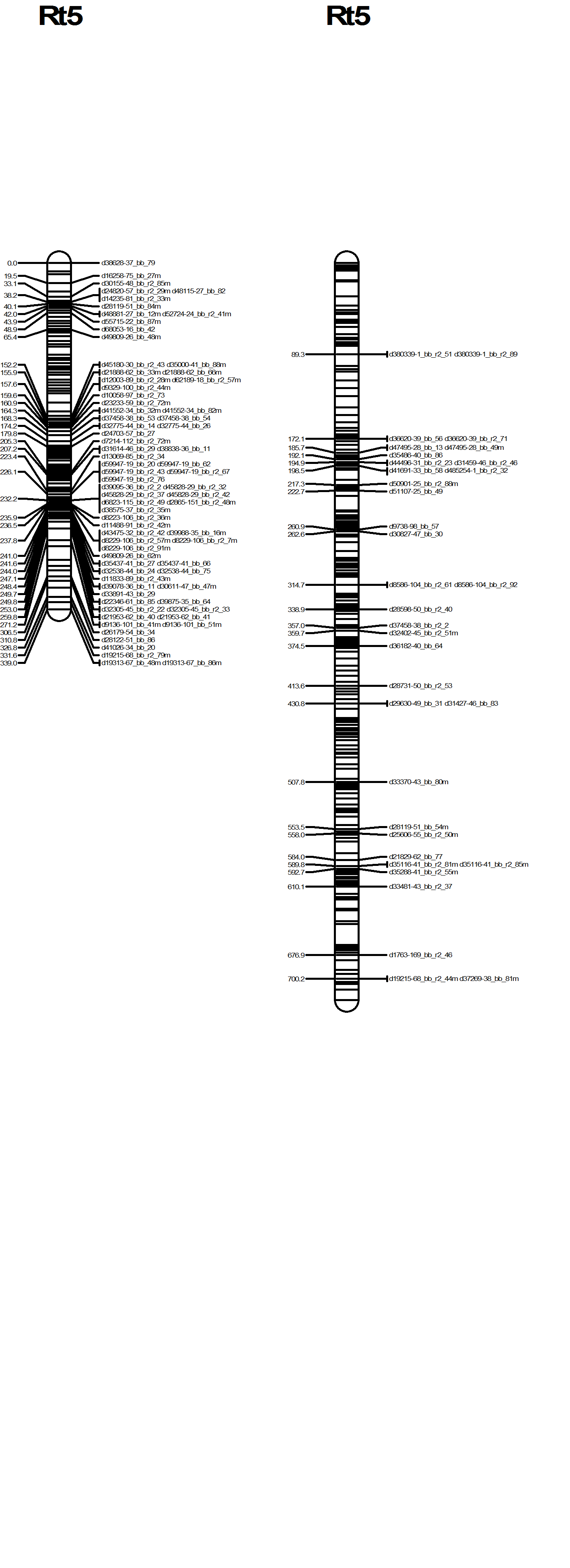


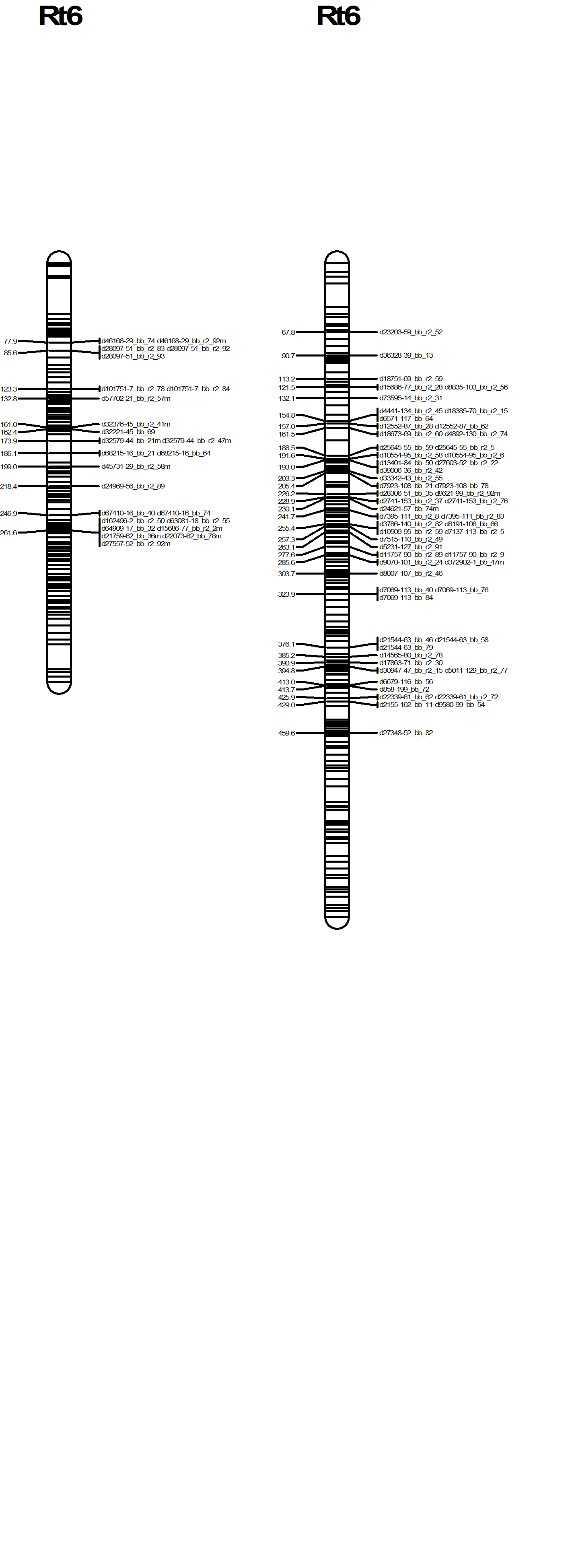


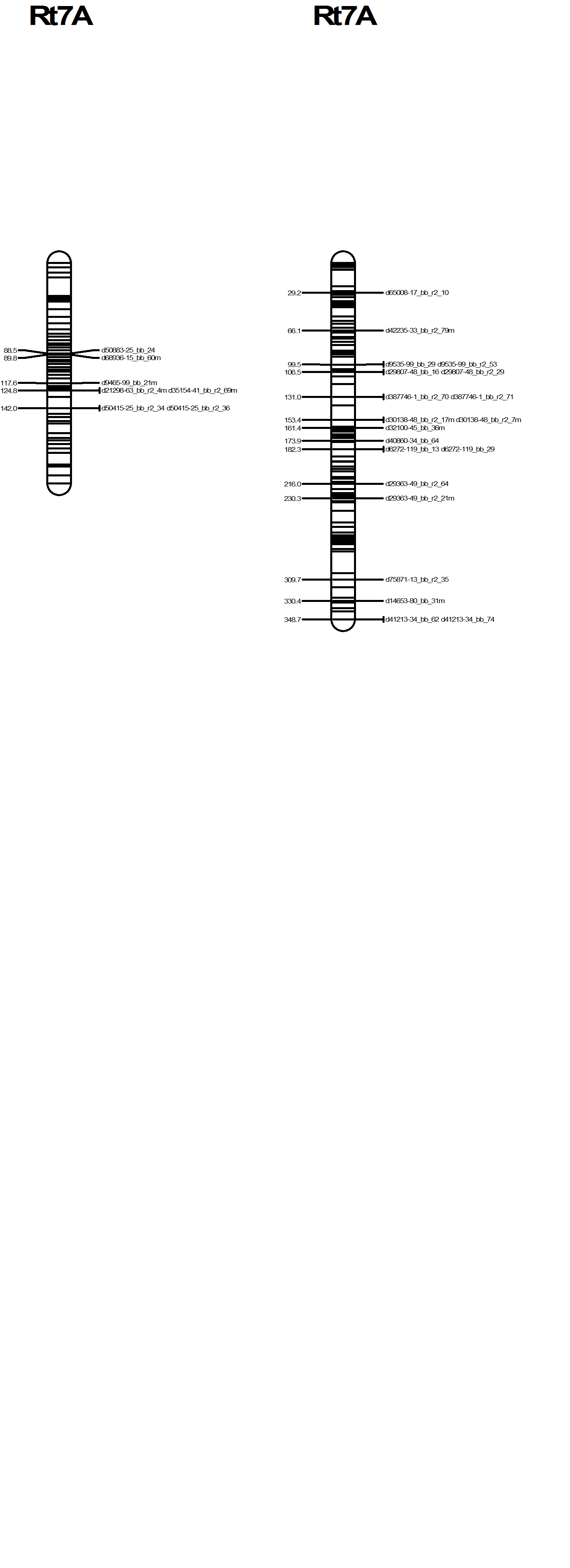


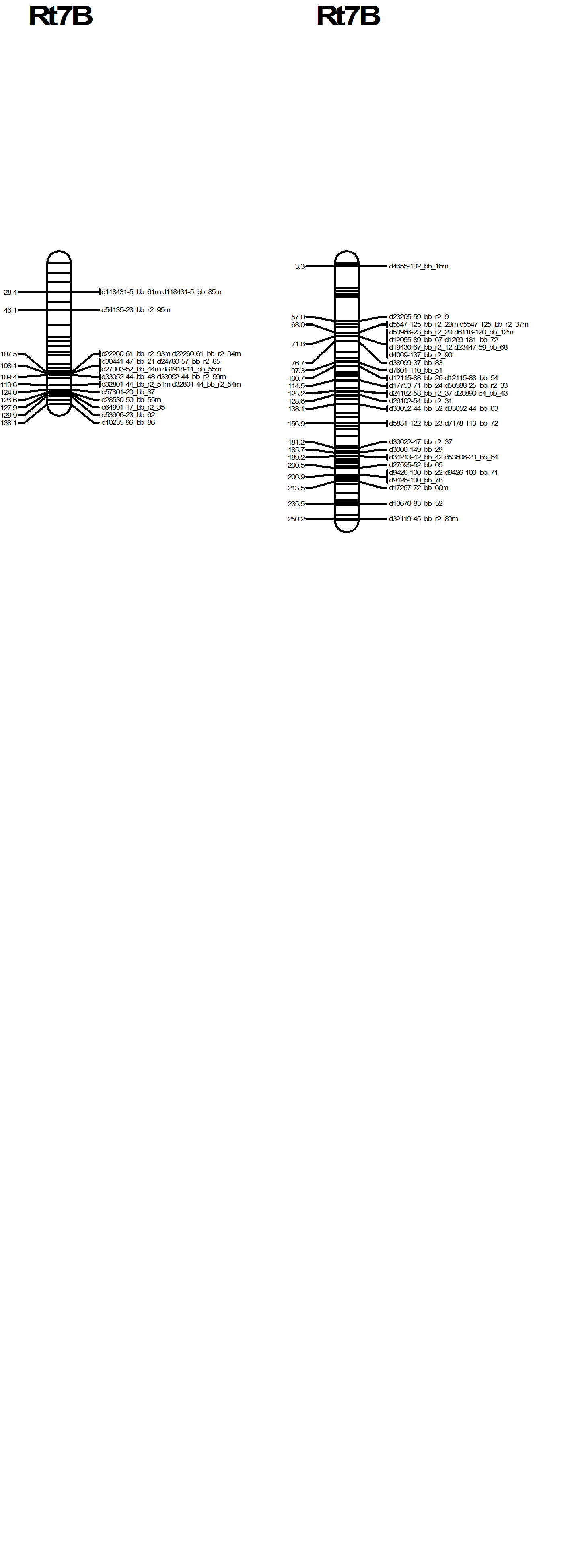


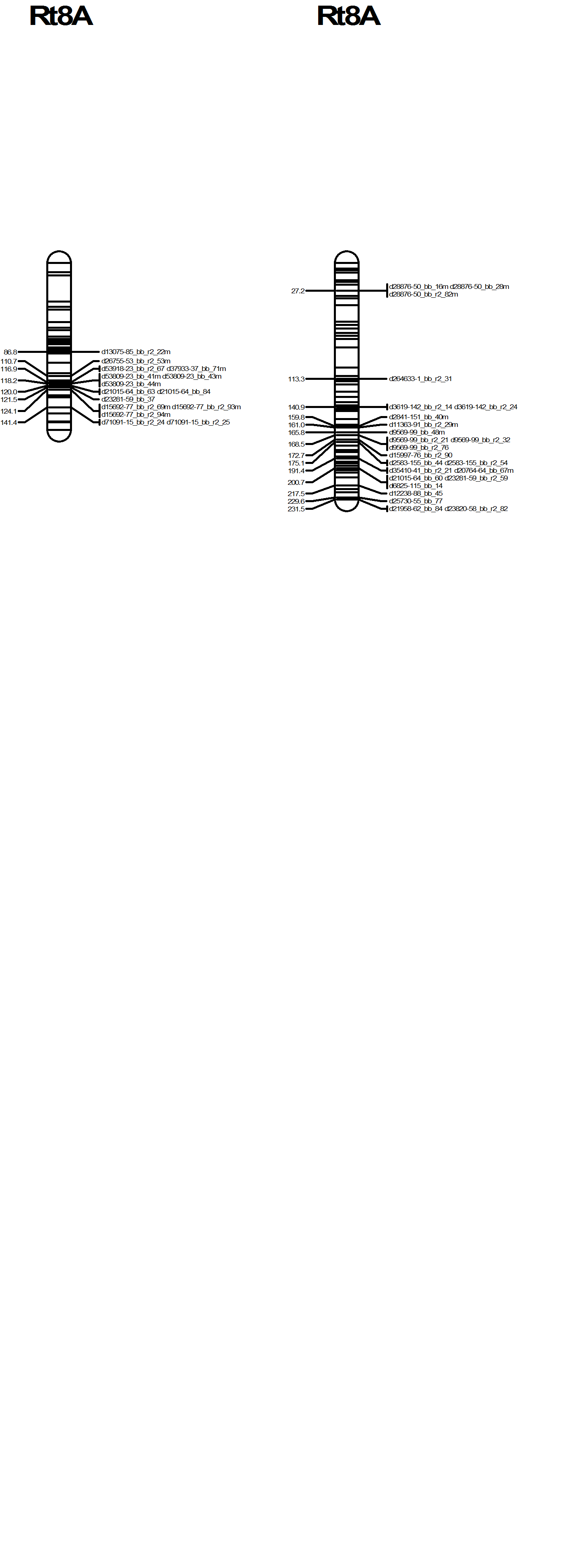


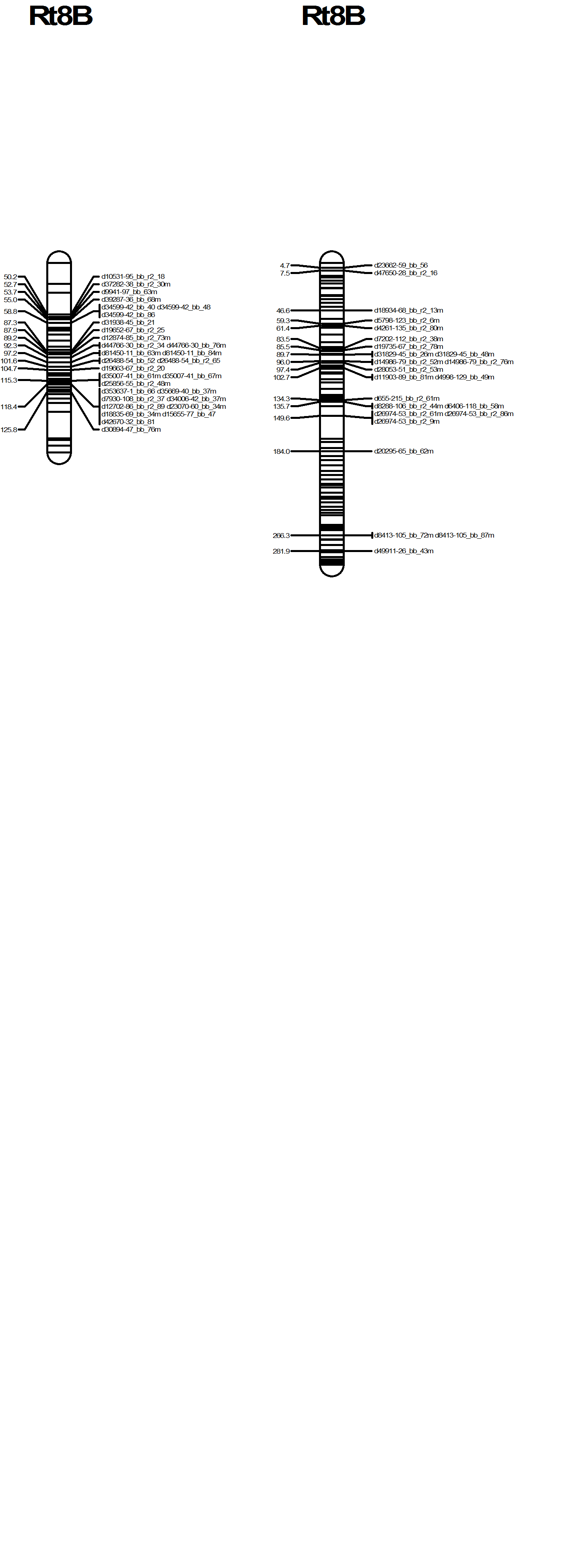


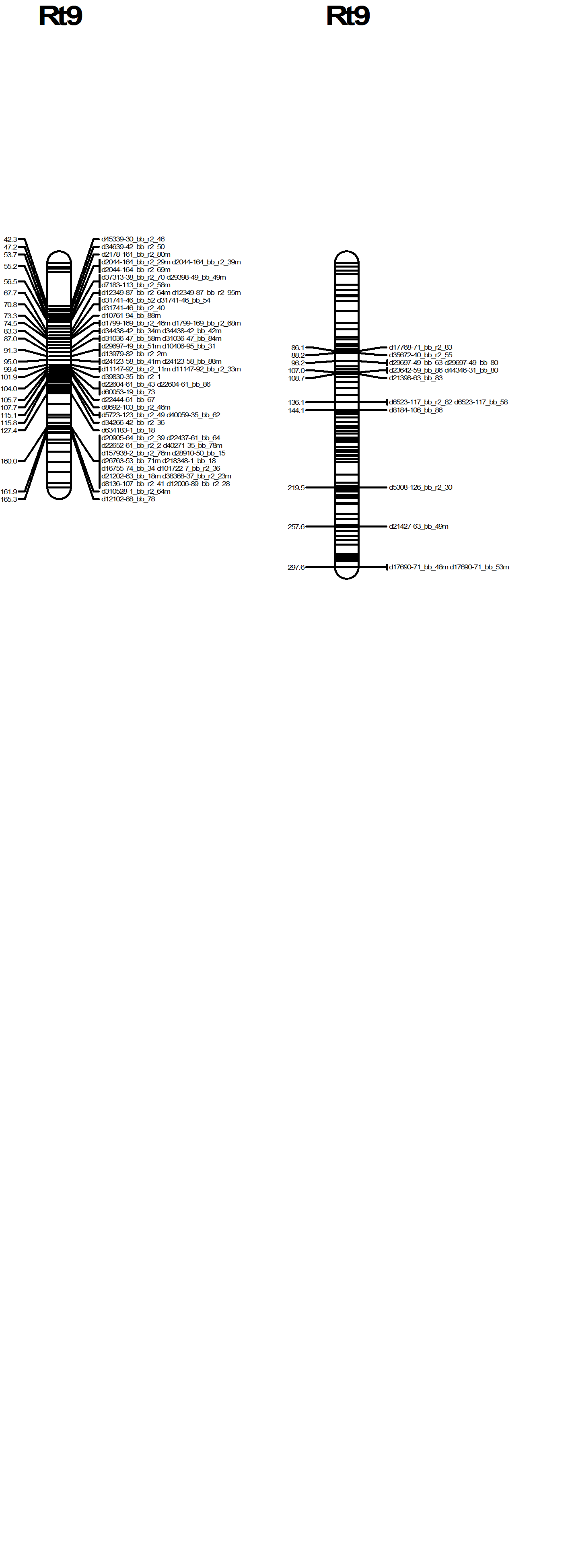


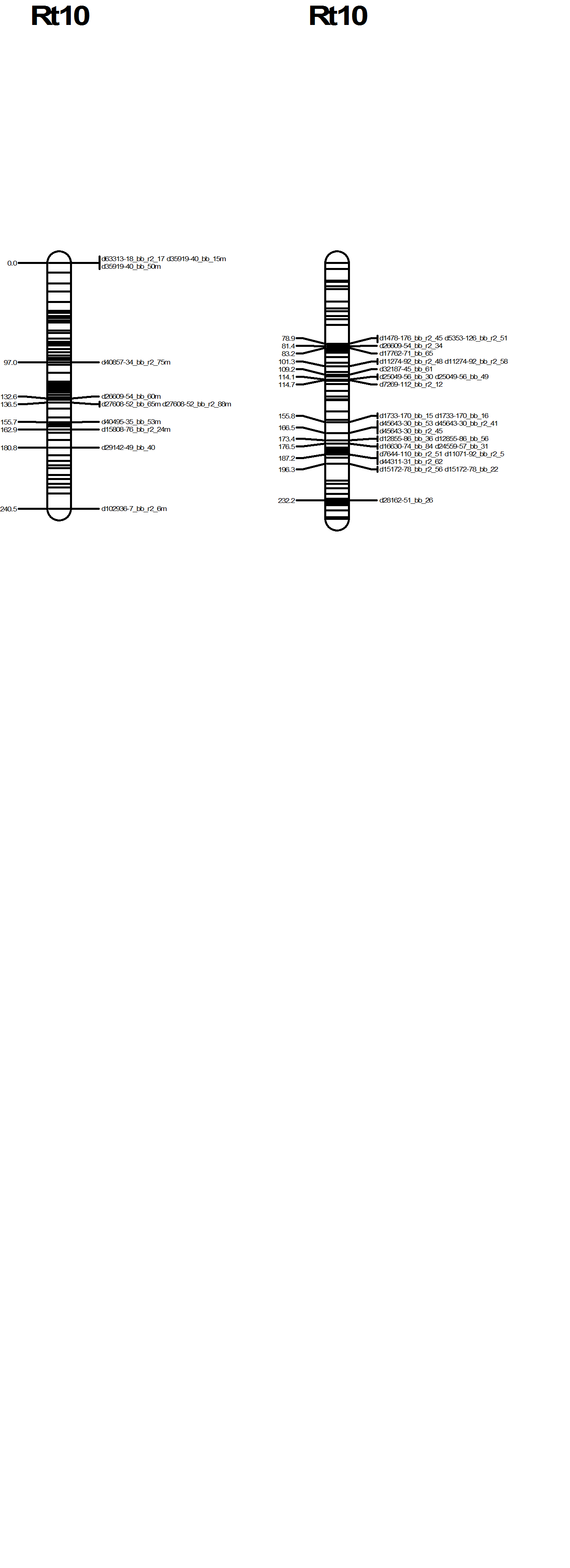

Supplement: Supplementary file 13 [file 637FileS6.docx]
